# Red Shrimp Are a Rich Source of Nutritionally Vital Lipophilic Compounds: A Comparative Study among Edible Flesh and Processing Waste

**DOI:** 10.3390/foods9091179

**Published:** 2020-08-26

**Authors:** Ramesh Kumar Saini, Min-Ho Song, Kannan R. R. Rengasamy, Eun-Young Ko, Young-Soo Keum

**Affiliations:** 1 Department of Crop Science, Konkuk University, Seoul 143-701, Korea; saini1997@konkuk.ac.kr (R.K.S.); tdasgtaasf@gmail.com (M.-H.S.); 2 Institute of Research and Development, Duy Tan University, Da Nang 550000, Vietnam; rengasamyrrajakannan@duytan.edu.vn; 3 Faculty of Environment and Chemical Engineering, Duy Tan University, Da Nang 550000, Vietnam; 4 Department of Food Science and Biotechnology of Animal Resources, Konkuk University, Seoul 143-701, Korea; key523@hanmail.net

**Keywords:** Argentine red shrimp, black tiger shrimp, white leg shrimp, cholesterol, α-tocopherol, polyunsaturated fatty acids (PUFAs), shrimp waste valorization

## Abstract

This study was aimed at comparatively analyzing the sterols, tocopherols and fatty acids from edible flesh and processing waste obtained from three shrimp species, utilizing rapid liquid chromatography (LC)-atmospheric-pressure chemical ionization (APCI)-tandem mass spectrometry (MS/MS) and gas chromatography-mass spectrometry (GC-MS). Results revealed the presence of significantly (*p* < 0.05) high proportions of health-beneficial omega-3 (n3) polyunsaturated fatty acids (PUFAs) in Argentine red shrimp (34.3% in waste and 38.2% in the flesh), compared to black tiger shrimp (16.5–24.2%) and whiteleg shrimp (13.2–22.6%). Among sterols, cholesterol was found most dominant, accounting in the range 349.4 (white shrimp flesh) to 559.3 µg/g fresh weight (FW) (black shrimp waste). Surprisingly, waste was found to contain a substantially higher amount of α-tocopherol, for instance, 21.7 µg/g FW in edible flesh and 35.3 µg/g FW in the waste of black tiger shrimp. The correlation analysis indicated that shrimp with low total contents of lipids might have higher proportions of health-beneficial long-chain (LC)-n3-PUFAs eicosapentaenoic (EPA) and docosahexaenoic acid (DHA). The fat quality indices, including the high ratios of hypocholesterolemic (h)/hypercholesterolemic (H) fatty acids, and lowest values of the atherogenic index (AI) and thrombogenic index (TI) indicated the health-beneficial potential associated with fat intake from red shrimp. Overall, a significant amount of health-beneficial compounds in edible flesh of studied shrimp confers its extraordinary nutritional benefits. Moreover, considering the richness of processing waste with these compounds, their valorization can be prompted.

## 1. Introduction

Shrimp are the most economically vital and globally traded commodity among crustaceans and all fish products [1]. According to the FAO statistics, 9.4 million tons of crustaceans (live weight), worth USD 69.3 billion were produced in 2018 [1]. Among them, whiteleg shrimp (*Penaeus vannamei*) alone accounted for 4.9 million tons of production (52.9% of total crustacean production) [1]. However, the marine capture production is dominated by Argentine red shrimp (*Pleoticus muelleri*) which accounted for 256 thousand tons of production (4% of the total 6 million tons of marine capture production of crustaceans) [1]. Shrimp are a key component of a Mediterranean diet, rich in protein, selenium, vitamin B12, vitamin E, omega-3 (n3) long-chain (LC)-polyunsaturated fatty acids (PUFAs), and astaxanthin, a potent antioxidant carotenoid [2]. Especially, shrimp are a rich source of health-beneficial eicosapentaenoic acid (EPA, n3) and docosahexaenoic acid (DHA, n3) that play the key roles in key regulating body homeostasis [3].

The domestic and industrial processing of shrimp involves the separation of head and carapace residues (waste) and muscles (edible flesh). Studies show that such processing generates 40–60% of food waste [4]. Given the high contents of nutritionally vital components in shrimp, their processing waste can be utilized to recover these nutrients, which can be utilized as health supplements [5]. Moreover, the valorization of processing this waste can solve the problem of its disposal. Additionally, it can generate surplus revenue which can significantly improve the economics of food production and processing.

Considering that a significant amount of waste is generated from shrimp processing, the present investigation was aimed at analyzing major sterols, tocols (Vit E; a sum of α-, β-, γ-and δ-tocopherol, and α-, β-, γ-, and δ-tocotrienol), and fatty acid from edible flesh and processing waste of three species of shrimp, utilizing modern liquid chromatography (LC)-atmospheric-pressure chemical ionization (APCI)-tandem mass spectrometry (MS/MS). Fatty acids were analyzed by gas chromatography (GC)-mass spectrometry (MS). The fatty acid composition data from edible flesh and processing waste were utilized to determine the fat quality characteristics. The results obtained herein are anticipated to contribute significantly to demonstrate the nutritional significance of shrimp, and valorization potential of shrimp processing waste.

## 2. Materials and Methods

### 2.1. Raw Materials, Chemicals, and Solvents

Three species of shrimp commonly consumed in Korea were procured form the Garak market, Songpa-gu, Seoul in October 2019 (Table 1). Two kg of each species in the frozen stage were brought to the lab, defrosted in lukewarm water, and the head and carapace residues (termed as waste) were manually separated from the edible flesh. The edible flesh and waste were separately homogenized using a food processor, and 10 g portion was precisely aliquoted to 50 mL Teflon lined glass tube and stored at −20 °C until analysis.

Authentic standards of fatty acids methyl esters (FAMEs, 37 mix CRM47885), tocols (mix of α-, β-, γ-and δ-tocopherol, and α-, β-, γ-, and δ-tocotrienol), and sterols, including 24α-ethyl cholesterol, 24α-methyl cholesterol, cholesterol, and 5-α-cholestan-3β-ol (internal standard) were obtained from Merck Ltd., Seoul, South Korea. All organic solvents used for extraction were of high-pressure liquid chromatography (HPLC) grade obtained from Daejung Chemicals & Metals Co., Ltd., Korea.

### 2.2. Extraction of Major Lipophilic Compounds

**Figure 1:**
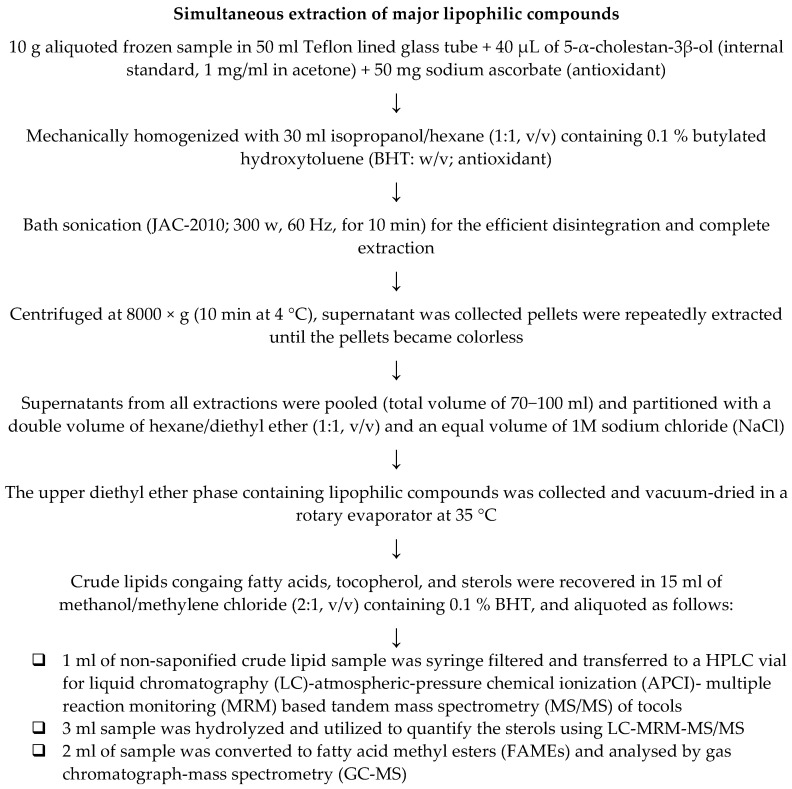
Method for the simultaneous extraction of major lipophilic compounds.

**Figure 2:**
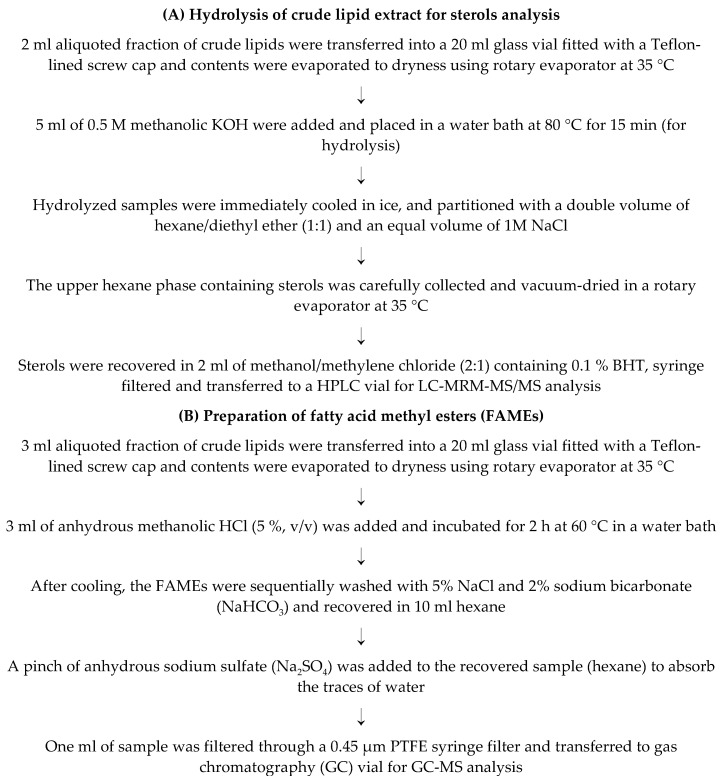
(**A**) Method for hydrolysis of crude lipid extract for sterols analysis, and (**B**) preparation of fatty acid methyl esters (FAMEs).

The major lipophilic compounds, including fatty acids, tocols, and sterols, were simultaneously extracted following the previous method [6,7,8] with minor modification. The details procedure is illustrated in Figure 1. The extracted crude lipids were aliquoted to three fractions, as illustrated in Figure 1 and utilized accordingly. Tocols were analyzed before hydrolysis as suggested by Cruz et al. [6]. Fraction 2 was hydrolyzed following the procedure of Cruz et al. [6] with minor modification (Figure 2A). The extracted crude lipids were converted to fatty acid methyl esters (FAMEs) (Figure 2B) and analyzed by gas chromatograph-mass spectrometry (GC-MS).

### 2.3. Analysis of Sterols, Tocols, and Fatty Acid Methyl Esters (FAMEs)

Sterols and tocols were quantified using liquid chromatography (LC)-atmospheric-pressure chemical ionization (APCI)- multiple reaction monitoring (MRM) based tandem mass spectrometry (MS/MS) studies as optimized recently [9]. The optimized instrumental parameters for the simultaneous analysis of sterols and tocols using LC-MRM-MS/MS are illustrated in Table 2. Similarly, the optimized values of collision energy (CE) and declustering potential (DP) of selected Q1 and Q3 MRM transitions for the simultaneous analysis of sterols and tocols using LC-MRM-MS/MS are given in Table 3.

Fatty acid methyl esters (FAMEs) were analyzed by gas chromatography (GC)-mass spectrometry (MS) utilizing a GC-2010 Plus Gas Chromatograph (Shimadzu, Kyoto, Japan) equipped with a QP2010 SE GC-mass spectrophotometer and an HP-5 column (Agilent; 30 m, 0.250 μm thick, and 0.25 mm ID). The injector port and ion source were maintained at 250 and 260 °C, respectively. Helium was used as a carrier gas. The thermal program followed 120–260 °C in 28 min (5 °C/min linear gradient) and held for 10 min. The FAMEs were precisely identified by comparing their retention time and fragmentation pattern with authentic standards [10].

### 2.4. Calculation of Fat Quality Indices

The fatty acid profile was used to determine several nutritional parameters of lipids, including ratios of PUFAs/saturated fatty acids (SFAs), PUFAs/monounsaturated fatty acids (MUFAs), and hypocholesterolemic (h)/hypercholesterolemic fatty acids [11,12]. Furthermore, the atherogenic index (AI) [11], and thrombogenic index (TI) [13] were calculated as the following equations:

(1) $$h/H=\frac{C18:1n9c+C18:2n6c+\;C20:3n6+C20:4n6+C20:5n3+\;C20:3n3+C20:4n3+\;C22:4n6+C22:5n6+C22:5n3+C22:6n3}{C14:0+C16:0}$$

(2) $$\mathrm{AI}=\frac{\left(4\times C14:0\right)+C16:0}{\mathrm{MUFAs}+\mathrm{FUFAs}}$$

(3) $$\mathrm{TI}=\frac{C14:0+C16:0+\;C18:0}{\left(0.5\times \mathrm{MUFAs}\right)+\left(0.5\times n6\;\mathrm{FUFAs}\right)+\left(3\times n3\;\mathrm{FUFAs}\right)+\left(\frac{n3\;\mathrm{PUFAs}}{n6\;\mathrm{PUFAs}}\right)}$$

### 2.5. Statistical Analysis and Quality Control

The samples were extracted in triplicates and analyzed separately. The results were analyzed using IBM SPSS statistics (version 25) employing a one-way analysis of variance (ANOVA), and homogenous subsets were determined (considering a significance level of 0.05) to separate the mean values of edible flesh and processing waste of black (black tiger), white (whiteleg), red (Argentine red) shrimp.

## 3. Results and Discussion

### 3.1. Fatty Acids Composition and Fat Quality Indices

**Figure 3:**
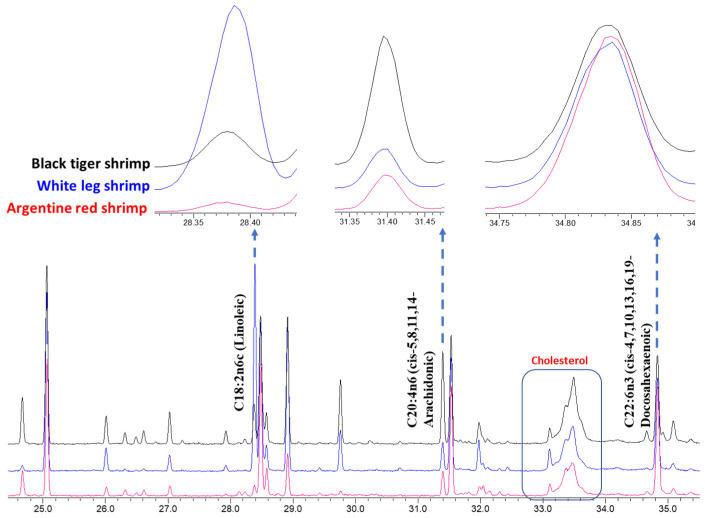
The representative gas chromatography (GC)-total ion chromatogram (TIC) profiles of fatty acid methyl esters (FAMEs) from the edible flesh of three shrimp species. The significant variation in fatty acid composition among black tiger, white leg, and Argentine red shrimp are shown.

**Figure A1:**
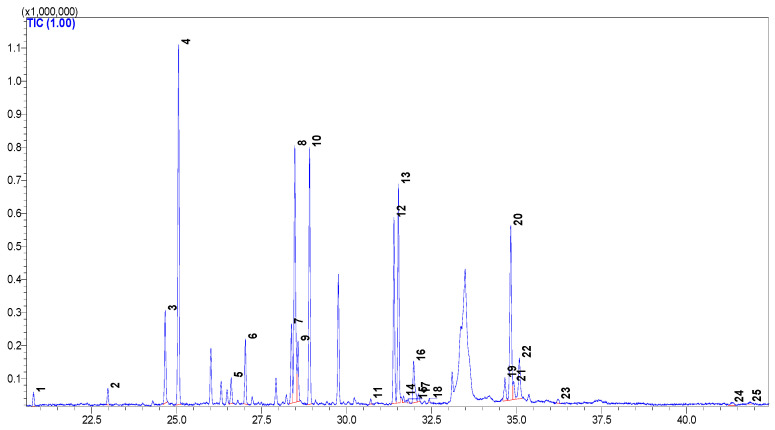
The representative GC-MS total ion chromatogram (TIC) profiles of fatty acid methyl esters (FAMEs) from black tiger shrimp (edible flesh). 1. C14:0 (Myristic), 2. C15:0 (Pentadecanoic), 3. C16:1n7c (cis-9; Palmitoleic), 4. C16:0 (Palmitic), 5. C17:1 (cis-10-Heptadecenoic), 6. C17:0 (Heptadecanoic), 7. C18:2n6c (Linoleic); 8. C18:1n9c (Oleic), 9. C18:1n7c (cis-11-octadecenoic), 10. C18:0 (Stearic), 11. C19:0 (Nonadecanoic), 12. C20:4n6 (cis-5,8,11,14-Arachidonic), 13. C20:5n3 (cis-5,8,11,14,17-Eicosapentaenoic), 14. C20:3n6 (cis-8,11,14-Eicosatrienoic), 15. C20:4n3 (cis 8,11,14,17-eicosatetraenoic; ETA), 16. C20:2 (cis-11,14-Eicosadienoic), 17. C20:3n3 (cis-11,14,17-Eicosatrienoic), 18. C20:0 (Arachidic), 19. C22:5n6 (cis- 4,7,10,13,16-docosapentaenoic; n6-DPA), 20. C22:6n3 (cis-4,7,10,13,16,19-Docosahexaenoic). 21. C22:4n6 (cis-7,10,13,16-Docosatetraenoic; DTA), 22. C22:5n3 (cis-7,10,13,16,19-docosapentaenoate), 23. C22:0 (Behenic), 24. C24:1n9 (Nervonic), and 25. C24:0 (Lignoceric).

**Figure A2:**
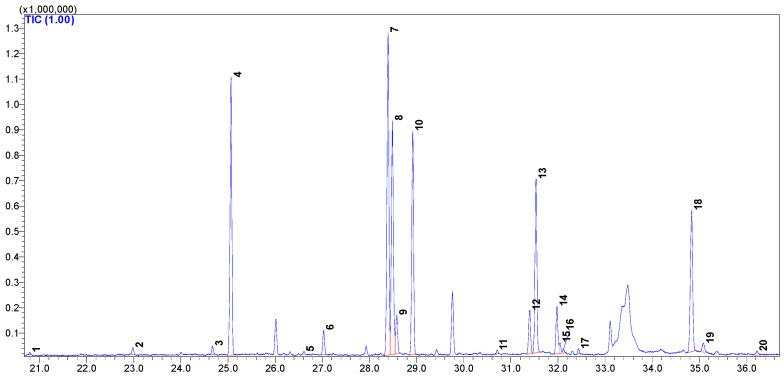
The representative GC-MS total ion chromatogram (TIC) profiles of fatty acid methyl esters (FAME) from whiteleg shrimp (edible flesh). 1. C14:0 (Myristic), 2. C15:0 (Pentadecanoic), 3. C16:1n7c (cis-9; Palmitoleic), 4. C16:0 (Palmitic), 5. C17:1 (cis-10-Heptadecenoic), 6. C17:0 (Heptadecanoic), 7. C18:2n6c (Linoleic), 8. C18:1n9c (Oleic), 9. C18:1n7c (cis-11-octadecenoic), 10. C18:0 (Stearic), 11. C19:0 (Nonadecanoic), 12. C20:4n6 (cis-5,8,11,14-Arachidonic), 13. C20:5n3 (cis-5,8,11,14,17-Eicosapentaenoic), 14. C20:2 (cis-11,14-Eicosadienoic), 15. C20:1n9 (cis-11-Eicosenoic), 16. C20:3n3 (cis-11,14,17-Eicosatrienoic), 17. C20:0 (Arachidic), 18. C22:6n3 (cis-4,7,10,13,16,19-Docosahexaenoic), 19. C22:5n3 (cis-7,10,13,16,19-docosapentaenoate), 20. C22:0 (Behenic).

**Figure A3:**
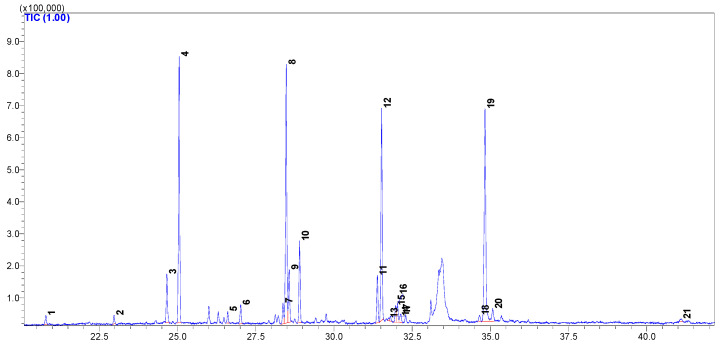
The representative GC-MS total ion chromatogram (TIC) profiles of fatty acid methyl esters (FAME) from Argentine red shrimp (Meat). 1. C14:0 (Myristic), 2. C15:0 (Pentadecanoic), 3. C16:1n7c (cis-9; Palmitoleic), 4. C16:0 (Palmitic), 5. C17:1 (cis-10-Heptadecenoic), 6. C17:0 (Heptadecanoic), 7. C18:2n6c (Linoleic), 8. C18:1n9c (Oleic), 9. C18:1n7c (cis-11-octadecenoic), 10. C18:0 (Stearic), 11. C20:4n6 (cis-5,8,11,14-Arachidonic), 12. C20:5n3 (cis-5,8,11,14,17-Eicosapentaenoic), 13. C20:3n6 (cis-8,11,14-Eicosatrienoic), 14. C20:4n3 (cis 8,11,14,17-eicosatetraenoic; ETA), 15. C20:2 (cis-11,14-Eicosadienoic), 16. C20:1n9 (cis-11-Eicosenoic), 17. C20:3n3 (cis-11,14,17-Eicosatrienoic), 18. C22:5n6 (cis- 4,7,10,13,16-docosapentaenoic; n6-DPA), 19. C22:6n3 (cis-4,7,10,13,16,19-Docosahexaenoic), 20. C22:5n3 (cis-7,10,13,16,19-docosapentaenoate), 21. C24:1n9 (Nervonic).

In the present study, 31 fatty acids were identified from edible flesh and waste of studied shrimp species (Table 4). In all the samples, palmitic (C16:0) and oleic acid (C18:1n9c) were found in the highest quantity (15.9–20.6% and 14.6–21.4%, respectively), followed by DHA (C22:6n3; cis-4,7,10,13,16,19), EPA (C20:5n3; cis-5,8,11,14,17), stearic (C18:0), and arachidonic acid (C20:4n6; cis-5,8,11,14). These six fatty acids together accounted for 57.7% (white shrimp waste) to 82.3% (red shrimp edible flesh) of total fatty acids. Among the identified fatty acids, the maximum variation was recorded for linoleic (C18:2n6c; 1.4% in red shrimp waste and 24.6% in red shrimp waste), arachidonic, and DHA (Figure 3). The GC-total ion chromatograms (TIC) of black tiger, whiteleg, Argentine red shrimp are were given in Figure A1, Figure A2 and Figure A3, respectively (Appendix A). Similarly, the mass spectrums of major identified fatty acids were given in Appendix B. In some samples (e.g., edible flesh of Argentine red shrimp), a fatty acid showed a similar fragmentation pattern, which was tentatively designated as C24:1 isomer (Appendix C).

Most crop seeds and vegetable oils, including canola, corn, soybean, and sunflower oils, are rich sources of n6 fatty acids in the form of linoleic acid with low proportions of n3 fatty acids, such as α-linolenic acid (C18:3n3; ALA) [14]. Meanwhile, health-beneficial LC-n3-PUFAs, such as EPA and DHA, are rare in most seeds commonly utilized for the extraction of vegetable oil [3]. Animals can synthesize n3 LC-PUFAs from common precursor ALA; however, this is with very low efficiency [3]. Thus, direct dietary or supplemental intake of LC-n3-PUFAs is considered beneficial to health [3].

Shrimp are well known to contain a significant amount of EPA, DHA, and other health-beneficial fatty acids [15,16,17]. The unusual presence of high proportions of linoleic acid and the composition of other fatty acids of white shrimp waste (*P. vannamei*) observed in the present study agree with previous reports [15,16]. In contrast, Sriket et al. [17] observed only a slight difference for the linoleic acid contents among edible flesh of black tiger shrimp (*P. monodon*; 13.0%) and white shrimp (*P. vannamei*, 15.6%). In the present investigation, we observed significantly (*p* < 0.05) low proportions of linoleic acid in black tiger (3.46% in waste and 3.79% in edible flesh), compared to whiteleg shrimp (24.6% in waste and 20.1% in edible flesh). Interestingly, in the present study, we observed that linoleic acid is compensated by palmitic, palmitoleic (C16:1n7c; cis-9), stearic, and arachidonic acid in the black tiger shrimp. Meanwhile, in the Argentine red shrimp, linoleic acid is compensated by EPA (14.1% in waste and 15.8% in edible flesh) and DHA (17.2% in waste and 20.2% in edible flesh) (Table 4). Furthermore, with the highest occurrence of EPA and DHA, the highest total amount of n3-PUFAs was recorded in Argentine red shrimp (34.3% in waste and 38.2% in edible flesh), with the highest ratio of n3/n6 PUFAs (4.03 in waste and 5.65 in edible flesh) (Table 5). Consumption of PUFAs in such proportions is highly beneficial to reduce the risk of CVD and many other chronic diseases [18]. Furthermore, among the studied shrimp, the lowest amount of total saturated fatty acids (SFAs) were recorded in Argentine red shrimp (25.2% in waste and 26.7% in edible flesh).

Fats with the PUFAs/SFAs ratio of greater than 0.45 are recommended for human consumption to minimize the risk of CVD and other chronic diseases [12]. In the present study, PUFAs/SFAs ratios ranged from 0.89 (black shrimp waste) to 1.70 (red shrimp waste) (Table 5), which falls within the recommendations. Moreover, the fats with lower AI and TI, and higher ratios of h/H fatty acids are recommended for minimizing the risk of CVD [11]. In the present study, the TI varied significantly among the studied shrimp species, and the lowest value of 0.18 was obtained in red shrimp (waste and edible flesh) (Table 5). Surprisingly, Rosa and Nunes [19] also recorded the TI of 0.18 in edible flesh of red shrimp (*Aristeus antennatus*) and Norway lobster (*Nephrops norvegicus*). In the present study, the AI ranged from 0.25 (white and red shrimp waste) to 0.42 (black shrimp waste) which is lower than reported from brown shrimp, *Crangon crangon* (AI of 1.34) [20]. Similarly, in the present investigation, the ratios of h/H fatty acids were recorded in the range of 2.03 (black shrimp waste) to 3.73 (white shrimp waste). Furthermore, considering the high ratios of h/H fatty acids, shrimp fats are health-beneficial, similar to duck meat (h/H = 3.5), marine fish fillets (h/H = 3.1), and common carp fillets (h/H = 3.4) [12].

Marine species, including shrimp, are an excellent source of health-beneficial LC-n3-PUFAs (especially EPA and DHA) [21]. Furthermore, among the studied shrimp species, Argentine red shrimp are the richest source of n3-LC-PUFAs with the lowest amount of SFAs. Moreover, head and carapace residues are found to contain a similar amounts of these health-beneficial fatty acids, which are discarded during the processing.

### 3.2. Sterols and Tocols Composition

**Figure 4:**
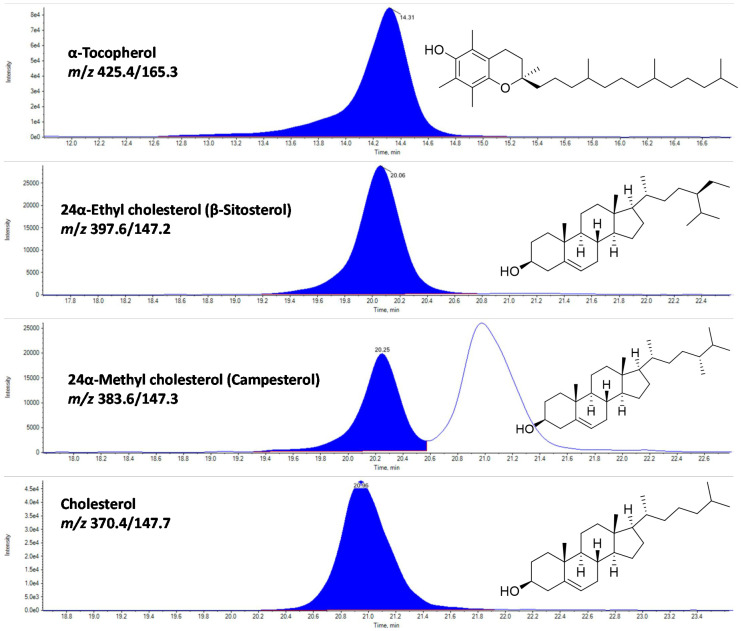
The representative LC-multiple reaction monitoring (MRM)-tandem mass spectrometry (MS/MS) chromatograms of α-tocopherol, and sterols in waste and edible flesh of three shrimp species.

**Figure 5:**
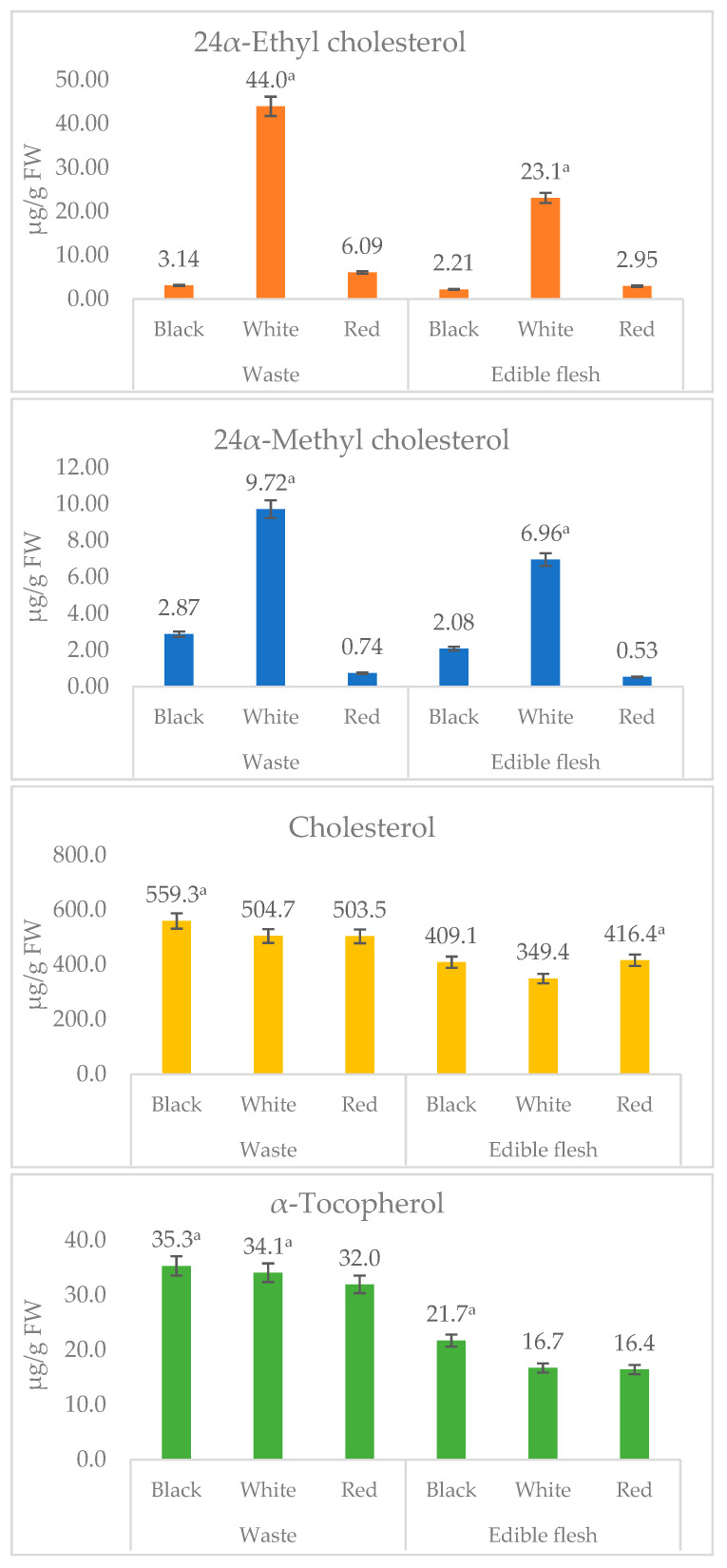
The content of α-tocopherol, and sterols in waste and edible flesh of three shrimp species. Values are mean ± standard deviation of three replicate determinations. ^a^ The mean value is significantly (*p* < 0. 05) highest among the processing waste or edible flesh obtained from black shrimp (black tiger), white (whiteleg), red (Argentine red) shrimp.

In the present study, three major sterols and α-tocopherol were identified using rapid LC-APCI-MRM based MS/MS analysis (Figure 4 and Figure 5). The representative LC-MRM-MS/MS chromatograms of sterols and α-tocopherol are given in Figure 4. Among waste and edible flesh of studied shrimp, cholesterol was found to be the most dominant, accounting for the range of 349.4 (white leg shrimp flesh) to 559.3 µg/g fresh weight (FW) (black shrimp waste). While other sterols, including 24α-ethyl cholesterol (β-sitosterol) and 24α-methyl cholesterol, were recorded in a small amount (Figure 5).

Similar to the edible flesh of other animals, such as egg, pork, and fish [2,22], shrimp are well known to contain a significant amount of cholesterol [23]. Tsape et al. [23] recorded 1440 and 5210 µg/g FW of cholesterol in muscles and cephalothorax of *Penaeus kerathurus*, respectively. Turan at al. [20] recorded the 1730 µg/g FW of cholesterol in edible flesh of brown shrimp, *C. crangon*. In contrast, 608–724 and 578–689 µg/g FW of cholesterol was recorded in edible flesh of red shrimp (*Aristeus antennatus*) and pink shrimp (*Parapenaeus longirostris*), respectively [19]. In contrast, in the present study, we recorded 349.4 (white shrimp flesh) to 559.3 µg/g FW (white shrimp waste) of cholesterol. The results of previous studies and the current study indicate that a significant variation is existing for cholesterol content among shrimp species.

In this study, shrimp waste and edible flesh samples were screened for α-, β-, γ-, and δ-tocotrienols and α-, β-, γ-, and δ-tocopherols; however, α-tocopherol was only recorded in a significant amount (16.4–35.3 µg/g FW). Surprisingly, waste was found to contain a significantly higher amount of α-tocopherol, for instance, 21.7 µg/g FW in edible flesh and 35.3 µg/g FW in the waste of black tiger shrimp.

A diet rich in cholesterol is considered a negative nutritional aspect, as excess intake of cholesterol may increase the risk of developing cardiovascular diseases (CVD) [22]. Moreover, cholesterol is known for the initiation of pathophysiological angiogenesis [24]. However, in recent years, several countries have withdrawn the upper limit (300 mg/day) of daily cholesterol intake, considering only a slight effect of dietary cholesterol on plasma cholesterol and overall impact on the risk of CVD among the healthy population [22,25]. Moreover, the results of the present study indicate that consumption of 100 g edible flesh of studied shrimps may provide only 34.9–41.6 mg cholesterol, which is under safe limits. Besides, shrimp are rich in health-beneficial LC-n3-PUFA and α-tocopherol, a key cellular antioxidant.

The correlation coefficients (r) between major lipophilic compounds (fatty acids, sterols, and α-tocopherol) recorded in studied shrimp species are given in Table 6. Correlation analysis shows that EPA and DHA are negatively correlated (r = −0.920 and −0.776, respectively) with crude lipids. The significantly higher proportions of linoleic acid in white leg shrimp lipids correlated positively with 24α-ethyl cholesterol (r = 0.952) and 24α-methyl cholesterol (r = 0.983), and negatively with EPA (r = −0.622) and DHA (r = −0.548). These observations suggest that shrimp with low total contents of lipids may have higher proportions of EPA and DHA.

### 3.3. Valorization Potential of Shrimp Processing Waste

In the present study, the processing of studied shrimp species generated 38.1% (black tiger shrimp) to 45.4% (Argentine red shrimp) waste (Table 1). Furthermore, the observations of an approximately equal amount of LC-n3-PUFAs, and significantly high amount of α-tocopherol in waste compared to edible flesh shows that nearly half of the amount of these beneficial nutritional compounds are eliminated during the domestic and industrial processing. Thus, we suggest that these processing waste can be utilized effectively for the recovery of these nutritional vital compounds. Furthermore, shrimp waste contains a significant amount of astaxanthin (e.g., 31–84 µg/g FW), chitin, and other nutritionally and commercially important nutraceuticals [4]. Moreover, α-tocopherol is generally supplemented with PUFAs rich lipids to enhance their oxidative stability [26,27]. In view of this, the natural presence of a significant amount of α-tocopherol in shrimp processing waste is advantageous. Thus, the simultaneous extraction of α-tocopherol, LC-n3-PUFA, and other nutraceuticals from shrimp waste can be promoted. The results of previous studies [4] and the present investigation provides support to create a foundation for the effective recovery of nutritional vital compounds from shrimp waste.

## 4. Conclusions

In summary, key nutritionally valuable lipophilic compounds were quantitatively analyzed in the edible flesh and processing waste utilizing the rapid and sensitive LC–MRM–MS/MS and GC–MS-based approaches. Among the studied shrimp, edible flesh and waste of Argentine red shrimp (*P. muelleri*) were found rich in health-beneficial LC-n3-PUFA. The fat quality indices, including the high ratios of PUFAs/SFAs and h/H fatty acids, and lowest values of AI and TI, also indicated the health-beneficial potential associated with fat intake from red shrimp. Surprisingly, higher contents of α-tocopherol and cholesterol are recorded in processing waste compared to edible flesh. A substantial amount of health-beneficial compounds in the edible flesh of studied shrimp confer its extraordinary nutritional benefits. Moreover, the recovery of these nutritional vital compounds from processing waste can be promoted for food fortification and supplement use.

## Figures and Tables

**Table 1:** List of shrimp samples procured for the study and the amount of waste generated from each species.

S/No.	Common Name	Scientific Name	Place of Origin	DOP *	% Waste
**1**	Black tiger shrimp	*Penaeus monodon*	Malaysia (cultivated)	June 2019	38.1
**2**	Whiteleg shrimp	*Penaeus vannamei* syn. *Litopenaeus vannamei*	Peru (cultivated)	June 2019	35.6
**3**	Argentine red shrimp	*Pleoticus muelleri*	Argentina (natural)	May 2019	45.4

* DOP: Date of packing.

**Table 2:** The optimized instrumental parameters for the simultaneous analysis of sterols and tocols using liquid chromatography-multiple reaction monitoring-mass spectrometry (LC-MRM-MS/MS).

	Instrumentation/Optimized Parameters
Mass spectrometer (MS)	Triple quadrupole MS (API 3200, Applied Biosystems-SCIEX, Framingham, MA, USA)
LC system	Exion LC™ system (SCIEX, USA)
MS operating mode	APCI positive
Column	C_30_ column (250 mm × 4.6 mm, 5 μm; YMC, Wilmington, NC, USA)
Column temperature	20 °C
Solvent system	A: Methanol/water (95/5, *v*/*v*) + 5 mM ammonium formate (AF; dissolved in water; B: tert-butyl methyl ether/methanol/water (90/7/3, *v*/*v*/*v*) + 5 mM AF
LC gradient elution	0–100% B for a total of 45 min analysis time, followed by a 5-min post-run for the column equilibrium
Flow rate	1 mL/min

**Table 3:** The optimized values of collision energy (CE) and declustering potential (DP) of selected Q1 and Q3 MRM transitions for the simultaneous analysis of sterols and tocols using LC-MRM-MS/MS.

S/No	Q1 (*m*/*z*)	Q3 (*m*/*z*)	Component Name	DP (V)	EP (V)	CE (eV)	CXP (V)	QL/QT	RT (min)
1	430.5	165.3	α-tocopherol (1)	65	10	40	5	QT	14.31
2	430.6	205.3	α-tocopherol (2)	65	10	40	5	QL	14.31
3	397.6	147.2	24α-ethyl cholesterol (1)	60	10	40	5	QT	20.06
4	397.6	161.4	24α-ethyl cholesterol (2)	60	10	40	5	QL	20.06
5	383.6	161.4	24α-methyl cholesterol (1)	70	10	40	5	QL	20.25
6	383.6	147.3	24α-methyl cholesterol (2)	70	10	40	5	QT	20.25
7	370.4	147.7	Cholesterol (1)	70	10	40	5	QT	20.96
9	370.4	161.7	Cholesterol (2)	70	10	40	5	QL	20.96
10	371.7	109.3	5-α-Cholestan-3-β-ol (1)	70	10	40	5	QT	21.92
11	371.7	135.3	5-α-Cholestan-3-β-ol (2)	70	10	40	5	QL	21.92

QL: Qualitative, QT: Quantitative, RT: Retention Time.

**Table 4:** The fatty acids composition of waste and edible flesh of three shrimp species.

S/No	RT	Component (Fatty Acid Methyl Esters)	Processing Waste			Edible Flesh		
			Black	White	Red	Black	White	Red
1	20.79	C14:0 (Myristic)	1.30 ± 0.01 ^a^	0.48 ± 0.01	0.62 ± 0.01	0.59 ± 0.06 ^a^	0.13 ± 0.04	0.65 ± 0.09 ^a^
2	22.98	C15:0 (Pentadecanoic)	1.20 ± 0.02 ^a^	0.52 ± 0.02	0.60 ± 0.06	0.75 ± 0.02 ^a^	0.39 ± 0.03	0.60 ± 0.04
3	24.58	C16:1n9c (cis-7; Hexadecenoic acid)	0.08 ± 0.05	0.15 ± 0.01	n.d.	n.d.	n.d.	n.d.
4	24.67	C16:1n7c (cis-9; Palmitoleic)	5.16 ± 0.19 ^a^	1.21 ± 0.02	3.28 ± 0.09	4.53 ± 0.09 ^a^	0.48 ± 0.08	3.47 ± 0.13
5	25.07	C16:0 (Palmitic)	20.6 ± 0.71 ^a^	15.9 ± 0.25	16.2 ± 0.16	16.8 ± 0.32	16.8 ± 0.04	18.5 ± 0.17 ^a^
6	26.61	C17:1 (cis-10-Heptadecenoic)	1.24 ± 0.05 ^a^	0.25 ± 0.04	0.76 ± 0.03	1.25 ± 0.06 ^a^	0.18 ± 0.03	0.74 ± 0.05
7	27.03	C17:0 (Heptadecanoic)	2.93 ± 0.03 ^a^	0.91 ± 0.02	1.15 ± 0.02	2.99 ± 0.05 ^a^	1.42 ± 0.02	1.35 ± 0.15
8	28.39	C18:2n6c (Linoleic)	3.46 ± 0.01	24.6 ± 0.21 ^a^	1.49 ± 0.05	3.79 ± 0.12	20.1 ± 0.11 ^a^	1.38 ± 0.10
9	28.49	C18:1n9c (Oleic)	14.9 ± 0.24	21.4 ± 0.33 ^a^	17.8 ± 0.20	14.6 ± 0.25	14.9 ± 0.16	18.7 ± 0.15 ^a^
10	28.58	C18:1n7c (cis-11-octadecenoic)	3.48 ± 0.10	3.17 ± 0.11	4.10 ± 0.09 ^a^	2.85 ± 0.22	2.33 ± 0.14	3.62 ± 0.08 ^a^
11	28.92	C18:0 (Stearic)	10.9 ± 0.14 ^a^	7.76 ± 0.04	5.61 ± 0.12	11.90 ± 0.32	13.5 ± 0.15 ^a^	5.66 ± 0.14
12	30.71	C19:0 (Nonadecanoic)	0.38 ± 0.00 ^a^	0.29 ± 0.03	0.21 ± 0.04	0.26 ± 0.10 ^a^	0.26 ± 0.05 ^a^	n.d.
13	31.40	C20:4n6 (cis-5,8,11,14-Arachidonic)	7.20 ± 0.12 ^a^	1.47 ± 0.03	4.23 ± 0.08	8.88 ± 0.08 ^a^	2.69 ± 0.13	3.46 ± 0.03
14	31.53	C20:5n3 (cis-5,8,11,14,17-Eicosapentaenoic)	7.47 ± 0.03	4.69 ± 0.03	14.1 ± 0.06 ^a^	10.1 ± 0.21	11.1 ± 0.10	15.8 ± 0.24 ^a^
15	31.70	C20:3n6 (cis-8,11,14-Eicosatrienoic)	0.42 ± 0.05	0.22 ± 0.05	0.41 ± 0.07	0.33 ± 0.09 ^a^	n.d.	0.20 ± 0.05 ^a^
16	31.81	C20:4n3 (cis 8,11,14,17-eicosatetraenoic; ETA)	0.24 ± 0.05	0.28 ± 0.06	0.53 ± 0.08 ^a^	0.34 ± 0.24	n.d.	0.28 ± 0.04
17	31.98	C20:2 (cis-11,14-Eicosadienoic)	5.12 ± 0.04 ^a^	4.42 ± 0.06	1.89 ± 0.11	2.98 ± 0.21 ^a^	2.94 ± 0.03 ^a^	1.28 ± 0.11
18	32.06	C20:1n9 (cis-11-Eicosenoic)	0.95 ± 0.00	1.58 ± 0.06	2.15 ± 0.05 ^a^	n.d.	0.58 ± 0.11	1.40 ± 0.05
19	32.13	C20:3n3 (cis-11,14,17-Eicosatrienoic)	0.40 ± 0.04	0.82 ± 0.04	1.08 ± 0.06 ^a^	0.51 ± 0.11	0.25 ± 0.06	0.80 ± 0.04 ^a^
20	32.44	C20:0 (Arachidic)	0.18 ± 0.09P	0.46 ± 0.02 ^a^	0.31 ± 0.03	0.21 ± 0.08 ^a^	0.32 ± 0.06 ^a^	n.d.
21	34.18	C21:0 (Henicosanoic)	n.d.	0.26 ± 0.07 ^a^	n.d.	n.d.	n.d.	n.d.
22	34.66	C22:5n6 (cis- 4,7,10,13,16-docosapentaenoic; n6-DPA)	0.77 ± 0.08	0.22 ± 0.02	0.47 ± 0.08	1.35 ± 0.10 ^a^	n.d.	0.46 ± 0.07
23	34.84	C22:6n3 (cis-4,7,10,13,16,19-Docosahexaenoic)	6.15 ± 0.33	6.48 ± 0.18	17.2 ± 0.19 ^a^	10.5 ± 0.38	10.6 ± 0.09	20.2 ± 0.28 ^a^
24	34.92	C22:4n6 (cis-7,10,13,16-Docosatetraenoic; DTA)	1.17 ± 0.03 ^a^	n.d.	n.d.	1.00 ± 0.11 ^a^	n.d.	n.d.
25	35.09	C22:5n3 (cis-7,10,13,16,19-docosapentaenoate)	2.19 ± 0.28 ^a^	0.89 ± 0.05	1.39 ± 0.15	2.72 ± 0.15 ^a^	0.65 ± 0.03	1.10 ± 0.29
26	35.73	C22:1n9 (Erucic)	0.00 ± 0.00	0.21 ± 0.07	0.83 ± 0.39 ^a^	n.d.	n.d.	n.d.
27	36.22	C22:0 (Behenic)	0.64 ± 0.08	0.49 ± 0.04	0.55 ± 0.11	0.28 ± 0.02	0.34 ± 0.01 ^a^	n.d.
28	38.74	C23:0 (Tricosanoic)	0.19 ± 0.07	0.20 ± 0.01	n.d.	n.d.	n.d.	n.d.
29	41.10	C24:1n9 (Nervonic)	0.36 ± 0.10	0.34 ± 0.08	1.56 ± 0.08 ^a^	n.d.	n.d.	0.36 ± 0.13
30	41.36	C24:1 isomer	0.53 ± 0.11	0.17 ± 0.02	1.57 ± 0.12 ^a^	0.22 ± 0.13 ^a^	n.d.	n.d.
31	41.86	C24:0 (Lignoceric)	0.39 ± 0.01 ^a^	0.20 ± 0.04	n.d.	0.20 ± 0.04 ^a^	n.d.	n.d.
32		Total SFAs	38.7 ± 0.60 ^a^	27.4 ± 0.13	25.2 ± 0.11	34.0 ± 0.62 ^a^	33.1 ± 0.06 ^a^	26.7 ± 0.53
33		Total MUFAs	26.7 ± 0.30	28.5 ± 0.45	32.0 ± 0.34 ^a^	23.5 ± 0.43	18.5 ± 0.10	28.3 ± 0.05 ^a^
34		Total PUFAs	34.6 ± 0.89	44.1 ± 0.31 ^a^	42.8 ± 0.44 ^a^	42.5 ± 1.01	48.4 ± 0.10 ^a^	45.0 ± 0.49
		n3 PUFA	16.5 ± 0.71	13.2 ± 0.17	34.3 ± 0.26 ^a^	24.2 ± 0.68	22.6 ± 0.06	38.2 ± 0.43 ^a^
		n6 PUFA	18.1 ± 0.18	30.9 ± 0.22 ^a^	8.50 ± 0.24	18.3 ± 0.41	25.8 ± 0.14 ^a^	6.77 ± 0.16
		Crude lipids (% DW)	5.95 ± 0.56	7.77 ± 0.68 ^a^	4.61 ± 0.54	4.60 ± 0.53	4.50 ± 0.50	3.65 ± 0.57

Values are mean ± standard deviation of percentages of the total fatty acids, from an average of three determinations. SFAs: total saturated fatty acids; MUFAs: total monounsaturated fatty acids; PUFAs: total polyunsaturated fatty acids; n.d.; not detected; RT: retention time; DW: dry weight. ^a^ The mean value is significantly (*p* < 0. 05) highest among the processing waste or edible flesh obtained from black (black tiger), white (white leg), red (Argentine red) shrimp.

**Table 5:** The fat quality indices of lipids obtained from waste and edible flesh of three shrimp species.

Indices	Processing Waste			Edible Flesh		
	Black	White	Red	Black	White	Red
PUFAs/SFAs	0.89 ± 0.04	1.61 ± 0.00	1.70 ± 0.03 ^a^	1.25 ± 0.05	1.46 ± 0.00	1.69 ± 0.05 ^a^
PUFAs/MUFAs	1.30 ± 0.05	1.55 ± 0.04 ^a^	1.34 ± 0.03	1.81 ± 0.08	2.62 ± 0.02 ^a^	1.59 ± 0.01
n3/n6	0.91 ± 0.03	0.43 ± 0.01	4.03 ± 0.10 ^a^	1.32 ± 0.03	0.88 ± 0.01	5.65 ± 0.13 ^a^
h/H	2.03 ± 0.09	3.73 ± 0.06 ^a^	3.50 ± 0.04	3.11 ± 0.08	3.57 ± 0.02 ^a^	3.27 ± 0.06
AI	0.42 ± 0.02 ^a^	0.25 ± 0.00	0.25 ± 0.00	0.29 ± 0.01 ^a^	0.26 ± 0.00	0.29 ± 0.01 ^a^
TI	0.46 ± 0.02 ^a^	0.35 ± 0.00	0.18 ± 0.00	0.31 ± 0.01	0.33 ± 0.00 ^a^	0.18 ± 0.00

Values are mean ± standard deviation of percentages of the total fatty acids, from an average of three determinations. SFAs: total saturated fatty acids; MUFAs: total monounsaturated fatty acids; PUFAs: total polyunsaturated fatty acids; h/H: ratios of hypocholesterolemic (h)/hypercholesterolemic (H) fatty acids; AI: atherogenic index; and TI: thrombogenic index. ^a^ The mean value is significantly (*p* < 0.05) highest among the processing waste or edible flesh obtained from black (black tiger), white (whiteleg), red (Argentine red) shrimp.

**Table 6:** Correlation between major lipophilic compounds of studied shrimp species.

	C16:1	C16:0	C18:2n6c	C18:1n9c	C18:1n9t	C18:0	C20:4n6	C20:5n3	C20:2	C22:6n3	Crude Lipids	α-tocopherol	24α-EC	24α-MC	Cholesterol
C16:1	1.000														
C16:0	0.640	1.000													
C18:2n6c	−0.856	−0.476	1.000												
C18:1n9c	−0.393	−0.407	0.356	1.000											
C18:1n9t	0.487	0.184	−0.619	0.390	1.000										
C18:0	−0.100	0.137	0.321	−0.747	−0.854	1.000									
C20:4n6	0.845	0.438	−0.650	−0.720	0.020	0.373	1.000								
C20:5n3	0.141	0.000	−0.622	−0.135	0.342	−0.391	0.005	1.000							
C20:2	0.077	0.326	0.411	−0.099	−0.252	0.464	0.168	−0.928	1.000						
C22:6n3	0.071	−0.126	−0.548	0.164	0.473	−0.623	−0.161	0.952	−0.959	1.000					
Crude Lipids	−0.224	−0.119	0.622	0.473	−0.047	0.021	−0.258	−0.920	0.805	−0.776	1.000				
α-tocopherol	0.245	0.110	0.047	0.279	0.489	−0.236	0.057	−0.603	0.653	−0.511	0.750	1.000			
24α-EC	−0.810	−0.537	0.952	0.604	−0.371	0.046	−0.731	−0.631	0.383	−0.475	0.738	0.225	1.000		
24α-MC	−0.751	−0.379	0.983	0.346	−0.584	0.343	−0.562	−0.753	0.564	−0.676	0.741	0.187	0.949	1.000	
Cholesterol	0.457	0.361	−0.200	0.278	0.672	−0.389	0.132	−0.425	0.545	−0.330	0.594	0.939	−0.002	−0.055	1.000

EC: ethyl cholesterol, MC: methyl cholesterol.

## Appendix A

The representative GC-MS total ion chromatogram (TIC) profiles of fatty acid methyl esters (FAMEs).

## Appendix B

The GC-mass spectrum of major FAME identified in black, white, and red shrimp.

## Appendix C

The tentative identification of peak 25 and 26 as C24:1n9 (Nervonic acid; Argentine red shrimp waste).
